# Climate change and infectious diseases in the Arctic: establishment of a circumpolar working group

**DOI:** 10.3402/ijch.v73.25163

**Published:** 2014-09-30

**Authors:** Alan J. Parkinson, Birgitta Evengard, Jan C. Semenza, Nicholas Ogden, Malene L. Børresen, Jim Berner, Michael Brubaker, Anders Sjöstedt, Magnus Evander, David M. Hondula, Bettina Menne, Natalia Pshenichnaya, Prabhu Gounder, Tricia Larose, Boris Revich, Karsten Hueffer, Ann Albihn

**Affiliations:** 1 Arctic Investigations Program, Division of Preparedness and Emerging Infections, National Center for Emerging and Zoonotic Diseases, Centers for Disease Control & Prevention, Anchorage, AK, USA; 2 Arctic Research Centre (ARCUM), Umea University, Umeå, Sweden; 3 Division of Infectious Diseases, Umea University, Umeå, Sweden; 4 Office of the Chief Scientist, European Centre for Disease Prevention and Control (ECDC), Stockholm, Sweden; 5 Zoonoses Division Centre for Food-borne, Environmental & Zoonotic Infectious Diseases, Public Health Agency of Canada, Saint-Hyacinthe, QC, Canada; 6 Department of Epidemiology Research, Staten Serum Institute, Copenhagen, Denmark; 7 Division of Community Health Services, Alaska Native Health Consortium, Anchorage, AK, USA; 8 Department of Clinical Microbiology, Bacteriology, Umea University, Umea, Sweden; 9 Department of Clinical Microbiology, Virology, Umeå University, Umea, Sweden; 10 School of Public Affairs, Arizona State University, Phoenix, AZ, USA; 11 School of Geographical Sciences and Urban Planning, Arizona State University, Phoenix, AZ, USA; 12 Global Change and Health, WHO Regional Office for Europe, European Centre for Environment and Health, Rome, Italy; 13 Department of Infectious Diseases and Epidemiology, Rostov State Medical University, Rostov-on-Don, Russia; 14 Department of Public Health and General Practice, Faculty of Medicine, Norwegian University of Science and Technology, Trondheim, Norway; 15 Institute of Forecasting, Russian Academy of Sciences, Moscow, Russian Federation; 16 Department of Biology & Wildlife, Institute of Arctic Biology, University of Alaska Fairbanks, Fairbanks, AK, USA; 17 Department of Biomedical Sciences and Veterinarian Public Health, University of Agricultural Sciences and National Veterinary Institute, Uppsala, Sweden

**Keywords:** climate change, infectious diseases, Arctic region, circumpolar working group

## Abstract

The Arctic, even more so than other parts of the world, has warmed substantially over the past few decades. Temperature and humidity influence the rate of development, survival and reproduction of pathogens and thus the incidence and prevalence of many infectious diseases. Higher temperatures may also allow infected host species to survive winters in larger numbers, increase the population size and expand their habitat range. The impact of these changes on human disease in the Arctic has not been fully evaluated. There is concern that climate change may shift the geographic and temporal distribution of a range of infectious diseases. Many infectious diseases are climate sensitive, where their emergence in a region is dependent on climate-related ecological changes. Most are zoonotic diseases, and can be spread between humans and animals by arthropod vectors, water, soil, wild or domestic animals. Potentially climate-sensitive zoonotic pathogens of circumpolar concern include *Brucella spp., Toxoplasma gondii, Trichinella spp., Clostridium botulinum, Francisella tularensis, Borrelia burgdorferi, Bacillus anthracis*, *Echinococcus spp., Leptospira spp*., *Giardia spp., Cryptosporida* spp., *Coxiella burnetti*, rabies virus, West Nile virus, Hantaviruses, and tick-borne encephalitis viruses.

An international circumpolar working group of subject matter experts from public health and academic institutions has been established to assess the potential emergence and health impact of climate-sensitive infectious diseases in northern human and animal populations, and to identify activities that may minimize the risks of disease emergence. Proposed activities include:

1. Enhance the capacity to monitor potentially climate-sensitive infectious diseases that are likely to have the most impact on human and animal populations as well as arthropod vectors and animal reservoirs of climate-sensitive infectious diseases.
2. Determine baseline levels of infections by conducting seroprevalence surveys in both human and animal populations.
3. Determine baseline levels of disease-transmitting vectors (presence, population density, active season, etc.)
4. Conduct research into the relationship between weather, climate, ecologic change and infectious disease emergence to guide early detection and intervention.
5. Develop communication strategies targeting health care providers, public and animal health practitioners, indigenous communities and other stakeholders including the public at large on the impact of climate change and infectious diseases.
6. Expand and intensify networks that could facilitate greater inter-sectorial cooperation between human, animal and environmental professionals in the Arctic on the national and international levels.

## Reasons for concern

The Arctic, even more so than other parts of the world, warmed substantially over the 20th century, principally in recent decades. According to the most recent assessment by the Intergovernmental Panel on Climate Change (1), climate models predict continued warming in the coming decades, with even greater warming in the Arctic resulting in a mean increase between 1.5 and 5.8°C by 2100. With these projected mean global temperature increases, it is likely that the Arctic sea ice cover will continue to shrink and thin, the winters will warm more so than summers, the mean annual precipitation will increase, global glacier volume will decrease and the Northern Hemisphere spring snow cover will decrease during the 21st century (1). Continued melting of permafrost and sea ice is expected to augment river discharge and contribute to a rise in sea level by 1 m by 2100. Moreover, some predictions suggest that these changes will be accompanied by greater overall climate variability and an increase in extreme weather events (2).

The current level of warming in the Arctic has already brought about substantial ecological and socio-economic impacts, caused by the thawing of permafrost, flooding, shoreline erosion, storm surges, and loss of protective sea ice (3). In many Arctic communities, the physical infrastructure was built on permafrost. Weakening of this permafrost foundation will likely damage water intake systems and pipes, and result in contamination of community water supplies. Moreover, the failure of the foundation of access roads, boardwalks, water storage tanks, and wastewater treatment facilities can turn water distribution and wastewater treatment systems inoperable. In the US Arctic (Alaska), reduced access to treated water for hygiene has already been shown to result in increased hospitalizations for skin infections and respiratory tract infections such as pneumonia (4,5). A number of Alaskan villages are already threatened by relocation due to the failing of foundational support for houses, water systems and civil infrastructure (3,6).

A shift in the boundaries of climatically and geographically linked ecosystems (biomes) will result in new or changing habitats for plants, insects and animals with profound implications for human activity. It is expected that the ecology and epidemiology of infectious diseases will change as well. Climate and weather affect the distribution and risk of many vector-borne diseases globally, such as malaria, Rift Valley Fever, plague and dengue fever in more southern latitudes (7). Weather also impacts the distribution of food- and water-borne diseases and emerging infectious diseases, such as West Nile virus, hantavirus and Ebola haemorrhagic fever (8–12). However, less is known about the influence of climate change and the risk and distribution of infectious diseases in Arctic regions (13,14). Nevertheless, it is likely that the climate change impacts could result in changes of rates of respiratory, skin and intestinal infections, and many other conditions caused by bacterial, viral and parasitic agents (3,4,5,15,16,17) . Specifically, rising temperatures are expected to favour a northward expansion of boreal forest into the tundra, and of tundra into the polar desert (2). Increasing temperatures may thus shift the density and distribution of animal reservoirs and arthropod vectors which could affect human and animal health or cause a shift in the geographical range of disease caused by these agents (*Echinococcus spp*, *Francisella tularensis*, West Nile virus, Hantaviruses, tick-borne encephalitis viruses, *Borrelia burgdorferi*, arboviruses including Sindbis virus, California serogroup viruses) (13,14,18–21). A 2014 report from northern Alaska documented an increased risk among residents of Arctic communities of contracting infectious diseases transmitted by wildlife (22). Invasive bacterial infections are of special concern in the Arctic (23,24). Rising temperatures may allow reservoir animal species such as rodents to survive winters in larger numbers, increase in population size and expand their habitat range. Such shifts can favour the transmission of *Brucella spp., Toxoplasma gondii, Trichinella spp., Coxiella burnetti* and Puumala hantavirus to humans in more northern locations (17,18,25–28).

Many Arctic residents depend on subsistence hunting, fishing and gathering for food and a stable climate for food storage. Food storage methods often include above ground air-drying and smoking of fish and meat at ambient temperature, below ground cold storage on or near the permafrost, as well as fermentation. Changes in climate may prevent the proper drying of fish or meat, resulting in spoilage and increasing the risk of botulism. Similarly, loss of permafrost may result in spoilage of food stored below ground (3). Loss of these traditional food storage methods will also contribute to reduced food security for many Arctic communities (29,30).

Changes in global and Arctic climates may result in increased transport of persistent organic pollutants and other toxic metals to the Arctic due to shifts in agriculture (31). Bioaccumulating contaminants in animals can impact their survival, affect their immune responses, their possible role as reservoirs of zoonotic agents and pose a food-safety risk if they are animal species hunted for food (32,33).

The impact of climate change on both human and animal diseases in the Arctic has not yet been fully evaluated. But there is clear potential for climate change to shift the geographical distribution northwards and into Arctic regions of certain vector-borne- and parasitic diseases, many being zoonotic diseases.

## Existing networks

Throughout the world, the globally interconnected nature of ecosystems, animal and human health has been recognized and is the foundation of the “One Health” concept defined as “the collaborative effort of multiple disciplines – working locally, nationally, and globally – to attain optimal health for people, animals and the environment” (34,35). This concept is nowhere more apparent than in the Arctic and the circumpolar North where “One Health” can be operationalized for the future health of ecosystems, humans and wildlife internationally (36). Building on and expanding existing networks is an important aspect of implementing the One Health approach. Networks that focus on human health in the Arctic include the International Union for Circumpolar Health (www.iuch.net) and the Circumpolar Health Research Network (www.circhnet.org).

The Arctic Council (www.arctic-council.org) provides the opportunity for human health networks to participate in working groups that focus on the wider aspects of health and of the Arctic environment. Established in 1996, The Arctic Council is a Ministerial intergovernmental forum promoting cooperation, coordination and interaction between the 8 Arctic States (the US, Canada, Denmark/Greenland, Iceland, Norway, Sweden, Finland and the Russian Federation), including Arctic indigenous populations on common Arctic concerns such as sustainable development and environmental protection in the Arctic.

The scientific work of the Arctic Council is carried out in 6 working groups: The Arctic Contaminants Action Program (ACAP), the Arctic Monitoring and Assessment Program (AMAP), Conservation of Arctic Flora and Fauna (CAFF), Protection of the Marine Environment (PAME), Emergency Prevention Preparedness and Response (EPPR) and Sustainable Development Working Group (SDWG). The working groups conduct research and other activities in the areas of monitoring, assessing and preventing pollution in the Arctic, climate change, biodiversity conservation, emergency preparedness and response, sustainable development and assessment of living conditions of Arctic residents including human health. The human health activities of the Arctic Council primarily reside in the AMAP Human Health Assessment Group where activities have focused on assessing the impact of environmental pollutants on human health, and more recently within the SDWG Human Health Experts Group where priorities include food and water security, injuries, mental behavioural health, diet and nutrition, health care delivery health inequities, climate change and infectious diseases (37).

The joint efforts between Pan American Health Organization and the World Health Organization Regional Office for Europe resulted in the development of a set of resources to support effective and evidence-based action to protect health from climate change (38,39). The European Ministerial Commitment to Act on climate change and health will further contribute to developments in strengthening health systems to cope with climate change, where the early identification of disease risks is essential (39).

## The International Circumpolar Surveillance climate change and infectious disease working group

The International Circumpolar Surveillance of Emerging Infectious Diseases (ICS) is an Arctic Council, SDWG project that aims to link public health laboratories, institutes and academic centres for the purpose of monitoring and sharing information on infectious diseases of concern, collaborating on research and prevention and control activities across the circumpolar north (40,41). Working groups have been established for surveillance of invasive bacterial diseases and tuberculosis, and research working groups have been formed for diseases caused by *Helicobacter pylori*, and viral hepatitis.

An ICS Climate Change and Infectious Diseases working group was formed at a meeting in Copenhagen on September 19, 2011. Members included international subject matter experts from public health and academic institutions drawn largely from existing circumpolar human and animal health networks. The technical expertise covered by the group includes circumpolar national and international knowledge of infectious disease surveillance and research, as they relate to climate change both for human and animal health.

The purpose of the ICS Climate Change and Infectious Disease Working Group is to share information on climate-sensitive infectious diseases in the circumpolar North. The goal is to identify potential cross-border collaborative surveillance and research activities that would allow for the monitoring of climate-sensitive diseases that have the potential to emerge or re-emerge. The goal is also to work towards establishing a formal quantitative link between weather, climate and infectious disease occurrence/prevalence and geographic distribution. Focus areas for the working group include surveillance and research activities on zoonotic and other infectious diseases arising from climate-change-related damage to water and sanitation infrastructure or water sources impacted by melting permafrost, storm surges, flooding, as well as infectious diseases related to food safety and security, and infectious diseases transmitted by arthropod vectors.

Early discussions have identified several potentially climate-sensitive infectious diseases of broad circumpolar concern. These not only include the mainly food-borne diseases such as Brucellosis, Toxoplasmosis, Trichinellosis and Botulism but also the vector-borne diseases of tularaemia, West Nile fever and California serogroup viruses. However, in some regions of Sweden, Norway, Finland and the Russian Federation, haemorrhagic fever with renal syndrome, tick-borne encephalitis, Lyme disease and Sindbis virus are climate-sensitive infectious diseases of increasing priority. The potential re-emergence of anthrax associated with historic livestock burial sites is of special concern in northern regions of the Russian Federation (14,42).

However, it is also apparent that the risk to human and animal populations for many of these diseases is not known, largely because surveillance systems are inadequate, lacking sensitivity and specificity required to determine with any accuracy the incidence and prevalence rates of disease in these populations. It is likely that this is due to under-reporting or under-diagnosis due to the lack of diagnostic infrastructure, limited staff capacity in remote locations or logistical difficulties associated with remote specimen collection, handling and shipping. Baseline levels of infection in humans, animals and vectors are also unknown. More research is needed to establish up to date incidence and prevalence of infection, to better understand the ecology of endemic and (re-) emerging pathogens and assess the potential impact of climate on disease occurrence in both human and animal populations in Arctic regions. Another limitation is access to timely, accurate and high-resolution geocoded climatic and environmental data. Such data can be linked with epidemiologic surveillance data in order to establish potential associations between climatic/environmental drivers and infectious diseases.

From working group meetings conducted since 2011, the following activities have been proposed to assess the health impacts of climate-sensitive infectious diseases in northern human and animal populations in Circumpolar Regions:

1. Enhance the surveillance capacity to monitor climate-sensitive infectious diseases that are likely to have the most impact on human and animal populations.
  An initial step will be to conduct surveys to assess reportable climate-sensitive infectious diseases by Arctic country or northern region. This catalogue can then be used to conduct surveillance evaluations for those climate-sensitive infectious diseases with the greatest potential for impacting human or animal health. All facets of each surveillance system should be examined to determine the number of cases identified, case definitions used, data collection, analysis, reporting and distribution systems used, including feedback to those providing the data.
2. Determine baseline levels of infection by conducting seroprevalence surveys in both human and animal populations. Conduct a survey of available human and animal specimen banks in the circumpolar north. Results could be used to target communities, or regions for specific prospective serosurveys, risk factor analysis, and lead to the implementation of prevention and control outreach, education and communication activities.
  Conduct serological analysis of subsistence harvested animal samples, targeting animals used for food production. Rural communities harvest thousands of marine and terrestrial animals each year. Wildlife managers in every northern region also collect small numbers of animal samples. These animals could serve as a key sentinel population to allow assessment of trends in the prevalence of known zoonotic pathogens as well as the detection of newly emerging infections. This can be achieved by developing local community-based hunter killed wildlife sampling programs using filter paper blood collection systems. Blood eluted from filter paper can be tested for a wide variety of zoonotic pathogens as well as toxic metals and persistent organic contaminants (43).
  Establish quality assurance programs in antibody and toxic metals and persistent organic contaminants detection and quantitation to allow sharing of standardized data between countries.
3. Determine the baseline level of specific disease vectors (mosquitoes, ticks, midges, etc.), targeting known vectors and reservoirs (e.g. wild-birds, rodents). Survey known vector collections and sample vectors to study trends of vector distribution related to climate- and land use change. Undertake targeted studies to better understand how climate change can influence the geographic range or abundance of animal reservoirs or arthropod vectors or the length of transmission cycles.
4. Conduct research into the relationship between weather, climate and infectious disease emergence to guide early detection and intervention. Promptly investigate outbreaks that may be climate related.
  A source of climatic or environmental data could be the European Environment and Epidemiology (E3) Network developed by the European Centre for Disease Prevention and Control (ECDC) (44). A recent data repository has been developed that provides access to climatic/environmental geospatial data for epidemiologic analysis (https://e3geoportal.ecdc.europa.eu/). For example, a comprehensive survey/review of health data resources in a particular region can be prepared that could be paired with environmental data for quantitative estimation of the links between climate and disease. Wildlife and public health agencies may have old banked blood from species important as food sources and these may augment current collection efforts and give important historic perspectives as to any association with temperature trends over time.
5. Establish a communication strategy to reach out to indigenous communities, circumpolar countries and other organizations and agencies with wildlife and human health responsibilities. The development of consumption advisories should be done in consultation with affected communities. This can be facilitated through web-based resources that will include diseases that are climate-sensitive, together with known spatial distribution, prevalence rates, the climate linkage framework for each, links to research papers and data sets focusing on each, geographical data on known outbreaks, case studies, agencies, research teams conducting studies and responding to health concerns.
6. Expand networks to facilitate greater inter-sectorial cooperation between the health, veterinary and environmental sectors in the Arctic. The interconnected nature of ecosystem health and human and animal health has been recognized and is the foundation of the “One Health” concept. This concept is particularly pertinent in the Arctic, making the circumpolar north the place where “One Health” can be examined. For example, disease ecology and the potential impact of climate on disease occurrence in both human and animal populations in Arctic regions can be studied. The key to capitalizing on the “One Health” approach is to use and expand interdisciplinary networks to establish and integrated disease surveillance using human animal and environmental data to detect emergence of climate-sensitive infectious diseases in human and animal populations.

## Conclusions

Predicting the effects of on-going climate and land use change on the transmission and impact of infectious diseases especially zoonotic diseases in the north is challenging because there is a lack of knowledge on the prevalence, diversity and distribution of these agents in these regions. Specifically, there is little formal evidence of a quantitative link between weather, climate and infectious disease prevalence and geographic distribution.

To address this challenge, the ICS Climate Change and Infectious Disease Working Group was established to provide a forum for sharing information on climate-sensitive infectious diseases in Arctic regions. It aims to develop strategies to assess the potential for disease (re) emergence with climate change. These include (a) identifying the steps that can be taken to improve surveillance, (b) determining baseline levels of infection, (c) conducting research into the relationship between weather and climate on infectious diseases in the Arctic and (d) establishing a communication strategy for data and information sharing about northern human and animal populations.

The working group provides the opportunity to bring together human, animal and environmental health professionals from other Arctic Council Working Groups, such as CAFF, PAME, and other networks and organizations, such as the WHO, CDC and ECDC, to share information on activities related to the impact of environmental change on human and animal health, and to provide a forum for identifying ideas and areas of common interest and collaboration at the local regional and circumpolar levels.
